# Supplementary material for: Enriched binocular experience followed by sleep optimally restores binocular visual cortical responses in a mouse model of amblyopia
Source: Commun Biol. 2023 Apr 13;6:408. doi: 10.1038/s42003-023-04798-y (PMC10102075; doi:10.1038/s42003-023-04798-y)
Supplement: Supplementary file 2 — Supplementary Information [file 42003_2023_4798_MOESM2_ESM.pdf]

Supplementary Information

Enriched binocular experience followed by sleep optimally restores binocular visual cortical responses in a mouse model of amblyopia

Jessy D. Martinez[1], Marcus J. Donnelly[2], Donald S. Popke[2], Daniel Torres[1], Lydia G. Wilson[1], William P. Brancaleone[2], Sarah Sheskey[3], Cheng-mao Lin[3], Brittany C. Clawson[1], Sha Jiang[1], Sara J. Aton[1*]

[1] Department of Molecular, Cellular, and Developmental Biology, University of Michigan, Ann Arbor, MI, USA.

[2] Undergraduate Program in Neuroscience, University of Michigan, Ann Arbor, MI, USA

[3] Department of Ophthalmology and Visual Sciences, University of Michigan Medical School, Ann Arbor, MI, USA

*Corresponding Author: saton@umich.edu

This PDF file includes:

Supplementary Figures 1-7

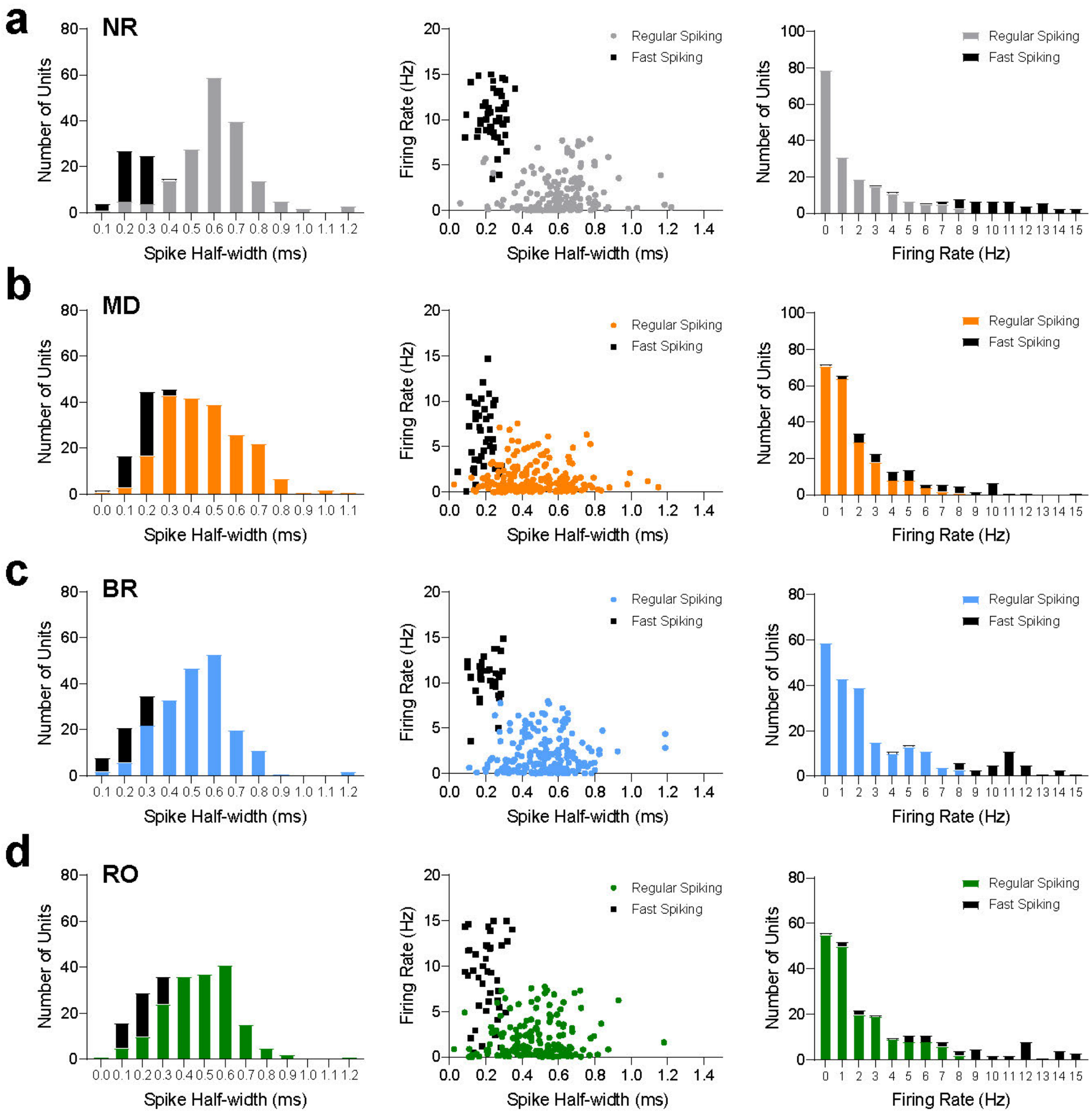

Supplementary Figure 1: Identification of fast-spiking (FS) interneurons and regular spiking (RS) neurons in NR, BR, and RO groups. (a-d) Distribution of spike half-widths (ms) for all recorded units shown in Figs. 1-2. FS interneurons' data are shown in black for all groups; regular spiking units are gray for NR **(a)**, orange for MD **(b)**, blue for BR **(d)**, and green for RO **(d)**. Comparisons of evoked firing rate vs. spike half-width yielded two distinct populations of recorded bV1 units corresponding to putative FS interneurons (black squares) and RS neurons (colored circles). Distribution of maximal evoked firing rates for all recorded units is also shown. Sample sizes: n = 5 mice/treatment group; Units: NR (n = 222), MD (n = 238), BR (n = 230), RO (n = 217).

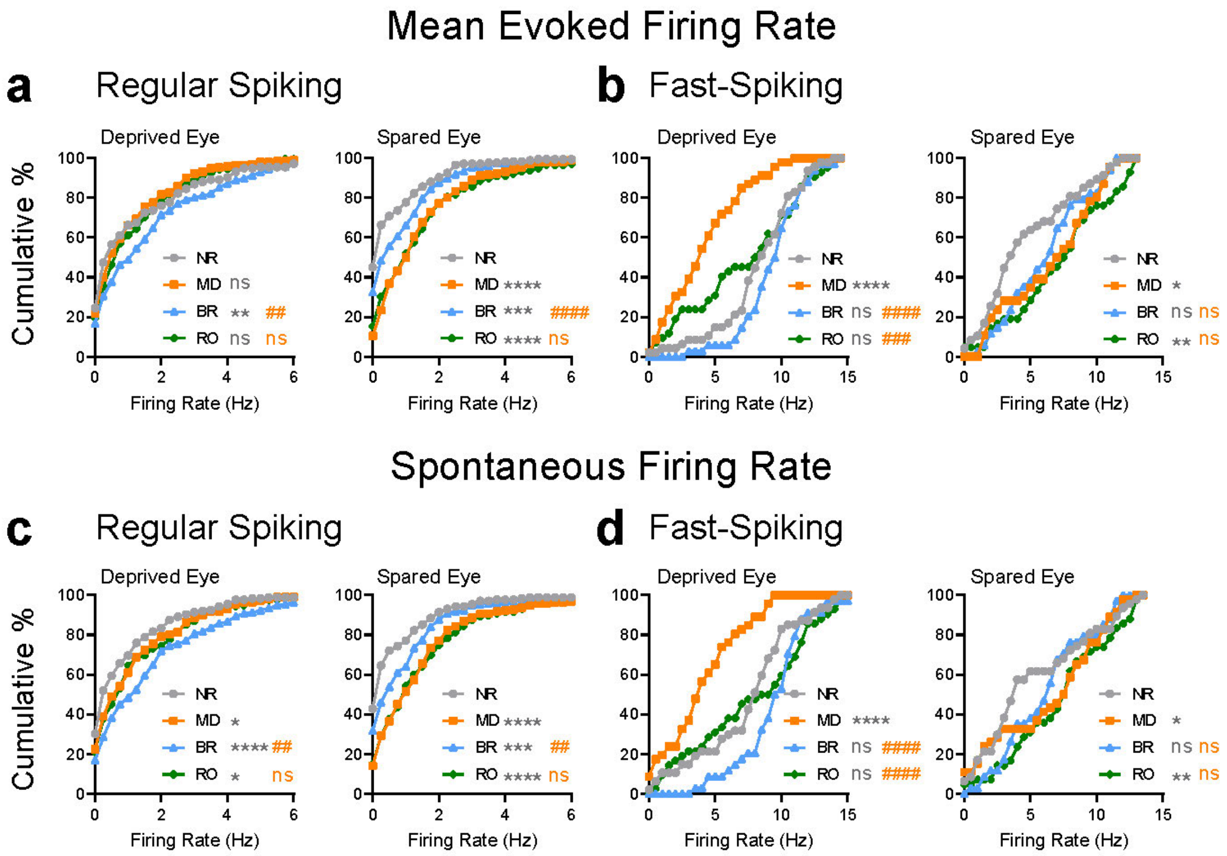

Supplementary Figure 2: Mean and spontaneous firing rate changes associated with MD, BR, and RO. (a) Cumulative distributions of deprived eye (DE) and spared eye (SE) mean visually-evoked firing rate responses (i.e. across all stimulus orientations) for bV1 regular spiking (RS) neurons. DE mean firing responses were unchanged after MD and RO, but increased after BR. MD enhanced SE mean firing responses. This enhancement was unaffected by RO and only partially reversed by BR. **(b)** Cumulative distributions of DE and SE mean visually-evoked firing rates for fast-spiking (FS) interneurons. DE responses were depressed after MD; this effect was reversed by both BR and RO. SE responses were enhanced after MD; this effect was partially reversed by BR, but not RO. **(c)** Cumulative distributions of RS neurons' DE and SE spontaneous firing rates (during presentation of a blank screen). MD enhanced DE spontaneous firing, and which was further enhanced by BR. **(d)** Cumulative distributions of FS neurons' DE (left) and SE (right) spontaneous firing rates. *, **, ***, and **** (gray) indicate $p < 0.05$, $p < 0.01$, $p < 0.001$, and $p < 0.0001$, respectively, K-S test vs. NR; ##, ###, #### (orange) indicate $p < 0.01$, $p < 0.001$, and $p < 0.0001$, respectively, K-S test vs. MD; ns indicates not significant. Sample sizes: $n = 5$ mice/treatment group; RS neurons: NR ($n = 175$), MD ($n = 192$), BR ($n = 196$), RO ($n = 175$); FS interneurons: NR ($n = 47$), MD ($n = 46$), BR ($n = 34$), RO ($n = 42$).

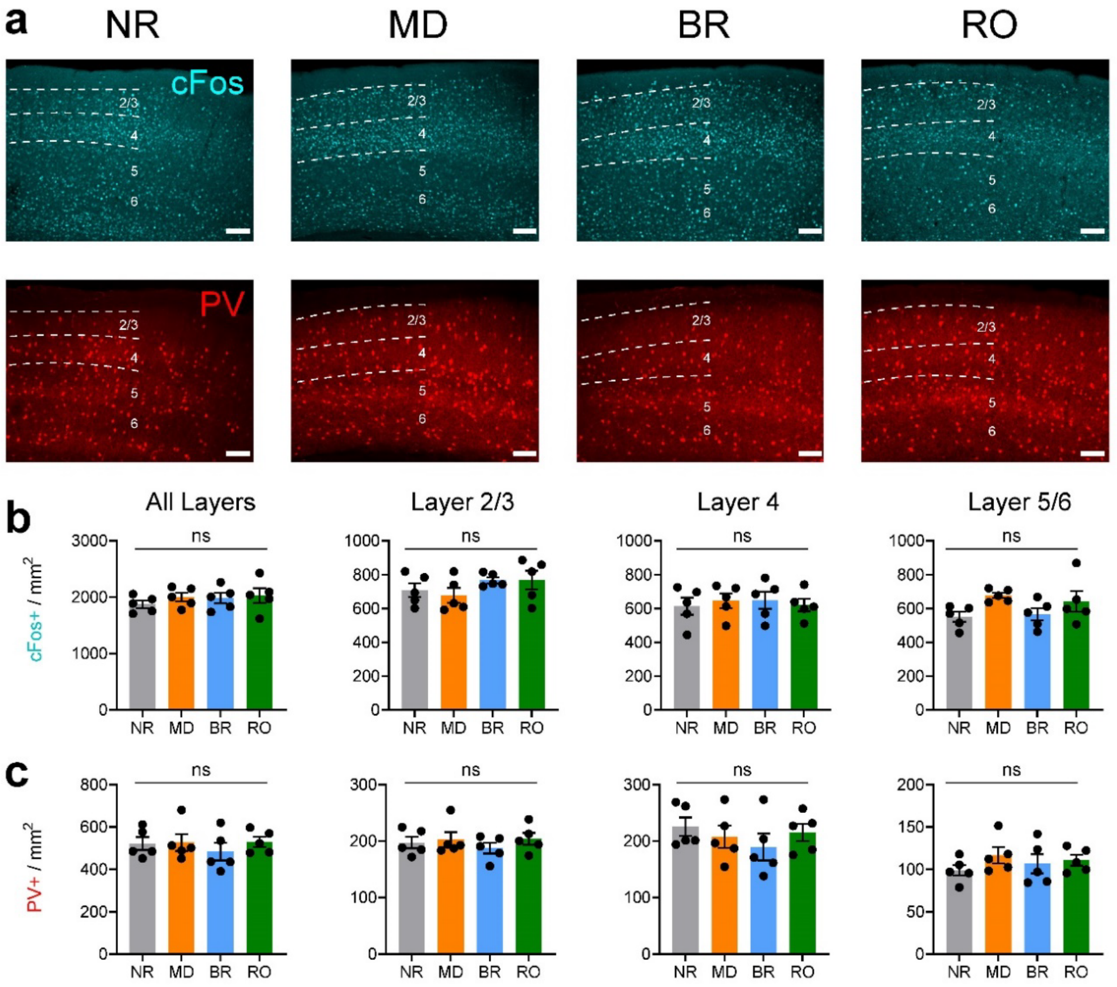

Supplementary Figure 3: cFos and PV expression are unchanged in primary auditory cortex (A1) after manipulations of visual experience. (a) Representative images of cFos (cyan) and parvalbumin (PV) [red] expression in A1 for the main treatment groups (n = 5 mice/treatment group). A1 regions were measured in the same brain sections, ipsilateral to bV1 measures (Fig 3). **(b)** No changes in cFos expression across NR, MD, and both visual experience groups were observed in total or across cortical layers. One-way ANOVA: F (3, 16) = 0.527, p = 0.669 for total cFos expression. cFos in bV1 layers 2/3, 4, and 5/6; one-way ANOVA for layers 2/3, 4, or 5/6, respectively: F (3, 16) = 1.17, p = 0.349, F (3, 16) = 0.153, p = 0.925, and F (3, 16) = 2.356, p = 0.110. **(c)** No changes in PV+ interneuron density groups were observed in total or across cortical layers. One-way ANOVA: F (3, 16) = 0.3867, p = 0.7641 for total PV+ density. PV+ interneuron density in bV1 layers 2/3, 4, and 5/6; one-way ANOVA for layers 2/3, 4, or 5/6, respectively: F (3, 16) = 0.456, p = 0.716, F (3, 16) = 0.631, p = 0.605, and F (3, 16) = 0.743, p = 0.541. Scale bar = 100 μm. Error bars indicate mean ± SEM.

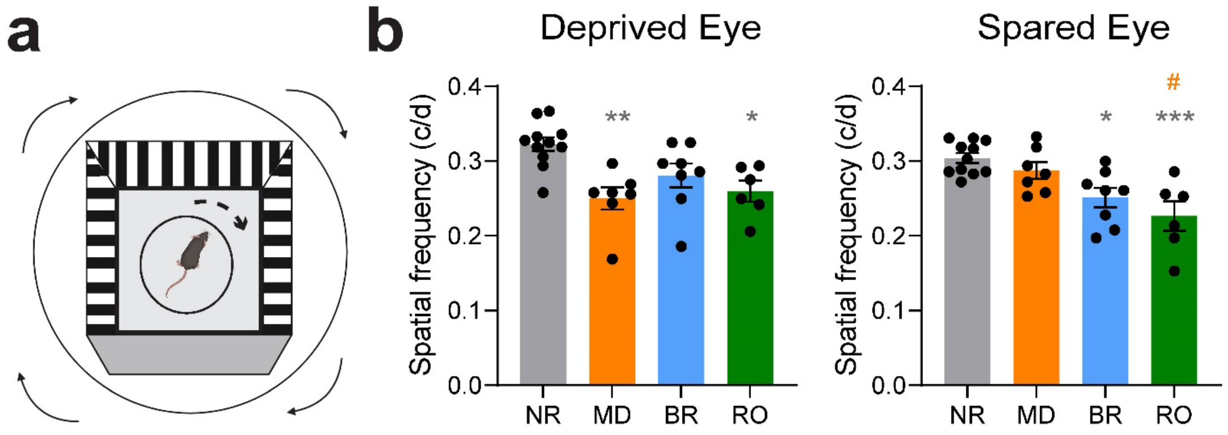

Supplementary Figure 4: Effects of MD, BR, and RO experience on visual acuity for the two eyes. (a) Optokinetic tracking responses to clockwise and counterclockwise drifting gratings of different spatial frequencies were presented to mice placed on an elevated platform in the center of the arena. **(b)** Threshold spatial frequency values for stimuli presented to the deprived eye (DE) and spared eye (SE) of mice in the four treatment groups. MD reduced DE acuity relative NR mice; DE acuity remained reduced after RO, but not BR. SE acuity was reduced after BR, and even further reduced after RO. One-way ANOVA: F (3, 28) = 6.74, p = 0.0014 for DE acuity and F (3, 28) = 8.64, p = 0.0003 for SE acuity, respectively. *, **, and *** (gray) indicate $p < 0.05$, $p < 0.01$, and $p < 0.001$, Tukey *post hoc* vs. NR; # (orange) indicates $p < 0.05$, Tukey *post hoc* vs. MD. Error bars indicate mean ± SEM. Sample sizes: NR (n = 11/mice), MD (n = 7/mice), BR (n = 8/mice), RO (n = 6/mice).

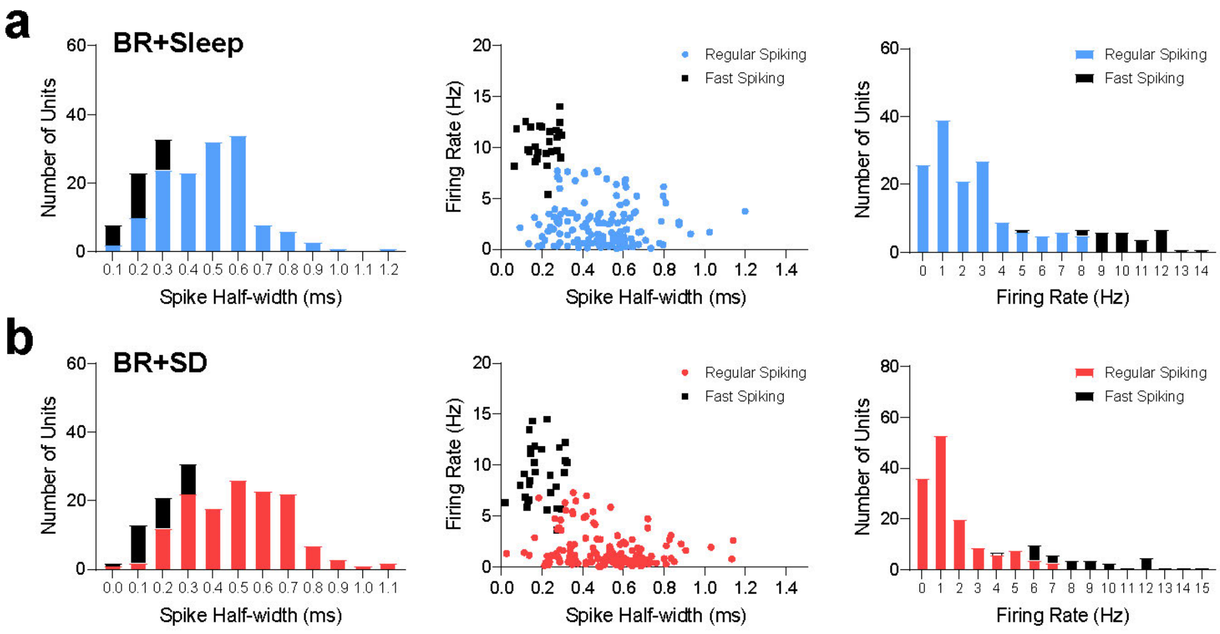

Supplementary Figure 5: Identification of fast-spiking (FS) interneurons and regular spiking (RS) neurons in BR+Sleep and BR+SD groups. (a-b) Distribution of spike half-widths (ms) for all recorded units shown in Figs. 4-5. FS interneurons' data are shown in black for all groups; regular spiking units are blue for BR+Sleep **(a)** and red for BR+SD **(b)**. Comparisons of evoked firing rate vs. spike half-width yielded two distinct populations of recorded bV1 units corresponding to putative FS interneurons (black squares) and RS neurons (colored circles). Distribution of maximal evoked firing rates for all recorded units is also shown. Sample sizes: *n* = 4 mice/treatment group; Units: BR+Sleep (*n* = 172), BR+SD (*n* = 169).

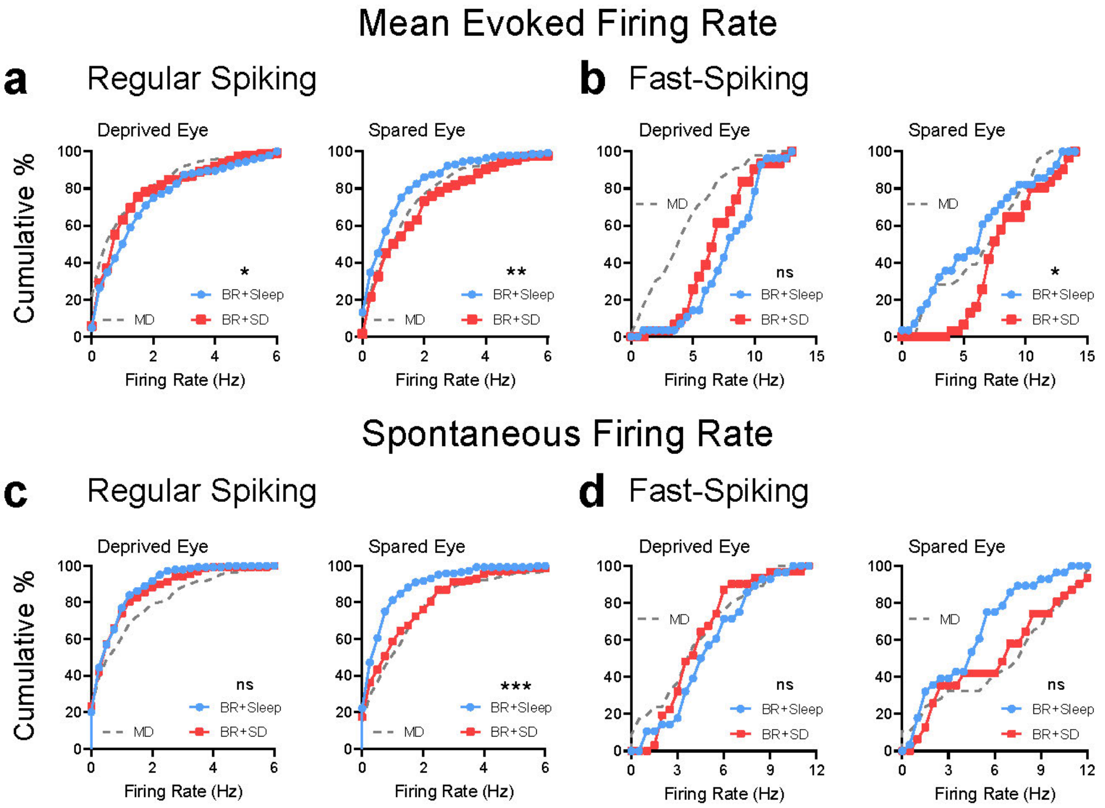

Supplementary Figure 6: Mean and spontaneous firing rate comparisons for BR+Sleep and BR+SD groups. (a) Cumulative distributions of deprived eye (DE) and spared eye (SE) mean visually-evoked firing rate responses (i.e. across all stimulus orientations) for bV1 regular spiking (RS) neurons. Similar to maximal visually-evoked firing at the preferred stimulus orientation (Fig. 5) mean DE responses were significantly decreased and SE responses were significantly increased in BR+SD mice, relative to responses recorded from BR+Sleep mice. **(b)** Cumulative distributions of DE and SE mean visually-evoked firing rate responses for bV1 fast-spiking (FS) interneurons. No changes in DE responses were observed. SE responses were significantly higher BR+SD mice compared to BR+Sleep counterparts. **(d)** Cumulative distributions of RS neurons' DE and SE spontaneous firing rates (during presentation of a blank screen). While DE spontaneous activity was unaffected, SE spontaneous activity was significantly lower in BR+Sleep mice. *, **, and *** indicate $p < 0.05$, $p < 0.01$, and $p < 0.001$, respectively, K-S test; ns indicates not significant. **(d)** FS interneurons' DE and SE spontaneous firing rates did not differ between BR+Sleep and BR+SD condition. Values for the MD-only condition (gray dashed lines) from Fig. 2 are shown for comparison. Sample sizes: n = 4 mice/treatment group; RS neurons: BR+Sleep (n = 144), BR+SD (n = 138); FS interneurons: BR+Sleep (n = 28), BR+SD (n = 31).

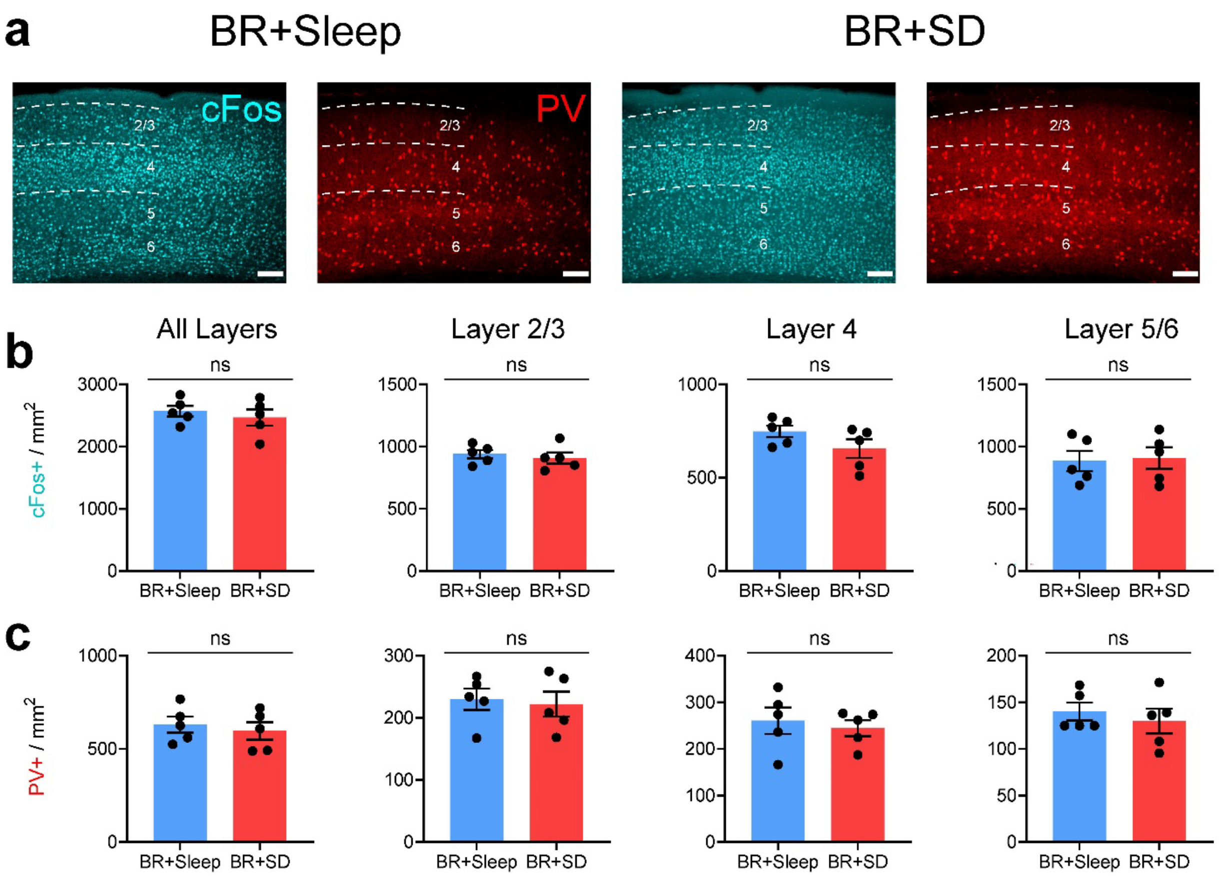

Supplementary Figure 7: cFos and PV expression in primary auditory cortex (A1) do not differ between BR+Sleep and BR+SD mice. (a) Representative images of A1 cFos (cyan) and parvalbumin (PV) [red] expression in BR+Sleep and BR+SD mice (n = 5 mice/treatment group), from the same brain sections shown in Fig. 6, and ipsilateral to measured DE expression in bV1. **(b-c)** No changes in cFos expression or PV+ interneuron density were observed between the groups. cFos expression: $p = 0.55$ (total), $p = 0.59$ (layer 2/3), $p = 0.15$ (layer 4), and $p = 0.83$ (layer 5/6). PV expression: $p = 0.61$ (total), $p = 0.78$ (layer 2/3), $p = 0.64$ (layer 4), and $p = 0.55$ (layer 5/6). Unpaired t-test. Scale bar = 100 μm.
