# Supplementary material for: Enriched binocular experience followed by sleep optimally restores binocular visual cortical responses in a mouse model of amblyopia
Source: Commun Biol. 2023 Apr 13;6:408. doi: 10.1038/s42003-023-04798-y (PMC10102075; doi:10.1038/s42003-023-04798-y)
Supplement: Supplementary file 3 — Description of Additional Supplementary Files [file 42003_2023_4798_MOESM3_ESM.pdf]

Description of Additional Supplementary Files

File name: Supplementary Data 1
Description: Source data underlying main figures.
