# Supplementary material for: Enriched binocular experience followed by sleep optimally restores binocular visual cortical responses in a mouse model of amblyopia
Source: Commun Biol. 2023 Apr 13;6:408. doi: 10.1038/s42003-023-04798-y (PMC10102075; doi:10.1038/s42003-023-04798-y)
Supplement: Supplementary file 5 — Reporting Summary [file 42003_2023_4798_MOESM5_ESM.pdf]

nature portfolio

Corresponding author(s): Aton, Sara J.

Last updated by author(s): Mar 29, 2023

Reporting Summary

Nature Portfolio wishes to improve the reproducibility of the work that we publish. This form provides structure for consistency and transparency in reporting. For further information on Nature Portfolio policies, see our Editorial Policies and the Editorial Policy Checklist.

Statistics

For all statistical analyses, confirm that the following items are present in the figure legend, table legend, main text, or Methods section.

n/a	Confirmed	
☐	☒	The exact sample size (*n*) for each experimental group/condition, given as a discrete number and unit of measurement
☐	☒	A statement on whether measurements were taken from distinct samples or whether the same sample was measured repeatedly
☐	☒	The statistical test(s) used AND whether they are one- or two-sided<br>*Only common tests should be described solely by name; describe more complex techniques in the Methods section.*
☐	☒	A description of all covariates tested
☐	☒	A description of any assumptions or corrections, such as tests of normality and adjustment for multiple comparisons
☐	☒	A full description of the statistical parameters including central tendency (e.g. means) or other basic estimates (e.g. regression coefficient) AND variation (e.g. standard deviation) or associated estimates of uncertainty (e.g. confidence intervals)
☐	☒	For null hypothesis testing, the test statistic (e.g. *F*, *t*, *r*) with confidence intervals, effect sizes, degrees of freedom and *P* value noted<br>*Give P values as exact values whenever suitable.*
☒	☐	For Bayesian analysis, information on the choice of priors and Markov chain Monte Carlo settings
☒	☐	For hierarchical and complex designs, identification of the appropriate level for tests and full reporting of outcomes
☒	☐	Estimates of effect sizes (e.g. Cohen's *d*, Pearson's *r*), indicating how they were calculated

Our web collection on statistics for biologists contains articles on many of the points above.

Software and code

Policy information about availability of computer code

Data collection	Plexon Omniplex software was used to collect all electrophysiological data. Leica Application Suite connected with a Leica SP8 confocal microscope was used to collect all imaging data. Cerebral Mechanics Optomotry was used to collect optokinetic tracking behavior data.
Data analysis	Electrophysiological data was organized using PlexUtil (version 4, Plexon) and spike sorting was done with Offline Sorter (version 4, Plexon). Further analyses were done with Neuroexplorer (version 5, Plexon). ImageJ (FIJI - ImageJ 1.52P) was used for all imaging analysis.

For manuscripts utilizing custom algorithms or software that are central to the research but not yet described in published literature, software must be made available to editors and reviewers. We strongly encourage code deposition in a community repository (e.g. GitHub). See the Nature Portfolio guidelines for submitting code & software for further information.

Data

Policy information about availability of data

All manuscripts must include a data availability statement. This statement should provide the following information, where applicable:

- Accession codes, unique identifiers, or web links for publicly available datasets
- A description of any restrictions on data availability
- For clinical datasets or third party data, please ensure that the statement adheres to our policy

Source data is provided with the manuscript in Supplementary Data. All other relevant raw data is available upon reasonable request from the corresponding author.

Human research participants

Policy information about studies involving human research participants and Sex and Gender in Research.

Reporting on sex and gender	N/A
Population characteristics	N/A
Recruitment	N/A
Ethics oversight	N/A

Note that full information on the approval of the study protocol must also be provided in the manuscript.

Field-specific reporting

Please select the one below that is the best fit for your research. If you are not sure, read the appropriate sections before making your selection.

☒ Life sciences ☐ Behavioural & social sciences ☐ Ecological, evolutionary & environmental sciences

For a reference copy of the document with all sections, see nature.com/documents/nr-reporting-summary-flat.pdf

Life sciences study design

All studies must disclose on these points even when the disclosure is negative.

Sample size	Power analyses were used to determine appropriate sample sizes.
Data exclusions	For electrophysiological recordings, mice whose recording electrode placement either could not be confirmed or was found not in binocular visual cortex were removed from study. For all studies, mice with impaired eye function (i.e. cataracts, infection) during recovery period were removed from study. In addition, mice that opened sutures during monocular deprivation period or reverse occlusion recovery period were also removed from study to remove mice with additional visual experience.
Replication	All attempts at data replication were successful.
Randomization	All mice were randomly assigned to experimental conditions.
Blinding	All data analyses were carried out in a bind manner. Electrophysiological and imaging data files were coded prior to analyses, which were carried out by experimenters blind to condition. Scorer for optokinetic behavior tracking studies was blind to treatment group during data collection and analyses.

Reporting for specific materials, systems and methods

We require information from authors about some types of materials, experimental systems and methods used in many studies. Here, indicate whether each material, system or method listed is relevant to your study. If you are not sure if a list item applies to your research, read the appropriate section before selecting a response.

Materials & experimental systems

n/a	Involved in the study	
☐	☒	Antibodies
☒	☐	Eukaryotic cell lines
☒	☐	Palaeontology and archaeology
☐	☒	Animals and other organisms
☒	☐	Clinical data
☒	☐	Dual use research of concern

Methods

n/a	Involved in the study	
☒	☐	ChIP-seq
☒	☐	Flow cytometry
☒	☐	MRI-based neuroimaging

Antibodies

Antibodies used	Immunohistochemistry was carried out using cFos (anti-rabbit; 1:1000; abcam ab190289) and parvalbumin (anti-mouse; 1:2000 Millipore, MAB1572). Secondary antibodies for cFos and parvalbumin were anti-rabbit Alexa Fluor 488 (1:200; Invitrogen, A11032) and anti-mouse Alexa Fluor 594 (1:200; Invitrogen, A11034).

Validation	Appropriate detection of cFos and parvalbumin antibodies were made using prior dilutions and experiments done by published work from our lab. Further validation on cFos antibody was done in a study measuring cFos detection in auditory cortex, a region on the same sections as the visual cortex highlighted in our study. We saw no changes in cFos across all experimental conditions in auditory cortex confirming that changes in cFos expression in visual cortex were due to experimental manipulation.

Animals and other research organisms

Policy information about studies involving animals; ARRIVE guidelines recommended for reporting animal research, and Sex and Gender in Research

Laboratory animals	For all experiments, male mice were used between postnatal day 28 and 38. C57BL/6J mice (Jackson Labs; Stock # 000664). Housing was maintained at 18-23°C and 40-60% relative humidity for all mice during all experiments. A 12:12 hour light:dark cycle was maintained with the exception of experiments described in Figures 4-6 and supplementary figures S5-S7 where we monitored mice under red light only for sleep deprivation studies.
Wild animals	No wild animals were used in the study
Reporting on sex	Because both synaptic structures and sleep architecture varies across the estrous cycle development, male mice were used for all studies.
Field-collected samples	No field collected samples were used in the study.
Ethics oversight	All mouse husbandry and experimental/surgical procedures described in this manuscript were reviewed and approved by the University of Michigan Internal Animal Care and Use Committee (IACUC).

Note that full information on the approval of the study protocol must also be provided in the manuscript.
